# N-3 Polyunsaturated Fatty Acids (PUFAs) Reverse the Impact of Early-Life Stress on the Gut Microbiota

**DOI:** 10.1371/journal.pone.0139721

**Published:** 2015-10-01

**Authors:** Matteo M. Pusceddu, Sahar El Aidy, Fiona Crispie, Orla O’Sullivan, Paul Cotter, Catherine Stanton, Philip Kelly, John F. Cryan, Timothy G. Dinan

**Affiliations:** 1 Department of Psychiatry and Neurobehavioural Science, University College Cork, Cork, Ireland; 2 APC Microbiome Institute, University College Cork, Cork, Ireland; 3 Teagasc, Moorepark, Cork, Ireland; 4 Department of Anatomy & Neuroscience, University College Cork, Cork, Ireland; Radboud University Medical Centre, NETHERLANDS

## Abstract

**Background:**

Early life stress is a risk factor for many psychiatric disorders ranging from depression to anxiety. Stress, especially during early life, can induce dysbiosis in the gut microbiota, the key modulators of the bidirectional signalling pathways in the gut-brain axis that underline several neurodevelopmental and psychiatric disorders. Despite their critical role in the development and function of the central nervous system, the effect of n-3 polyunsaturated fatty acids (n-3 PUFAs) on the regulation of gut-microbiota in early-life stress has not been explored.

**Methods and Results:**

Here, we show that long-term supplementation of eicosapentaenoic acid (EPA)/docosahexaenoic acid (DHA) (80% EPA, 20% DHA) n-3 PUFAs mixture could restore the disturbed gut-microbiota composition of maternally separated (MS) female rats. Sprague-Dawley female rats were subjected to an early-life stress, maternal separation procedure from postnatal days 2 to 12. Non-separated (NS) and MS rats were administered saline, EPA/DHA 0.4 g/kg/day or EPA/DHA 1 g/kg/day, respectively. Analysis of the gut microbiota in adult rats revealed that EPA/DHA changes composition in the MS, and to a lesser extent the NS rats, and was associated with attenuation of the corticosterone response to acute stress.

**Conclusions:**

In conclusion, EPA/DHA intervention alters the gut microbiota composition of both neurodevelopmentally normal and early-life stressed animals. This study offers insights into the interaction between n-3 PUFAs and gut microbes, which may play an important role in advancing our understanding of disorders of mood and cognitive functioning, such as anxiety and depression.

## Introduction

Stress, especially in early life has been identified as a cause of the disruption of this developmental pattern leading to a variety of disorders ranging from gastrointestinal disorders [1], to anxiety and depression [2]. In rodents, the maternal separation (MS) model is a well-known paradigm that induces brain-gut axis dysfunction [3]. The separated phenotype alters many components of the brain-gut axis throughout the body including the hypothalamic–pituitary adrenal (HPA) axis [3], the immune and neuroendocrine systems [4]. Growing evidence considers these abnormalities comorbid with changes in the gut microbiota [5–7] as well as crucial risk factors for the development of mental illnesses such as anxiety and depression [8, 9].

There is increasing evidence suggesting a bi-directional communication between the central nervous system (CNS) and the gut-microbiota which is recognized as the microbiome–gut–brain axis [10–12]. This communication is believed to influence the parallel development of both CNS and gut microbiota which can remarkably influence health and disease [13]. Emerging evidence has shown the involvement of the gut microbiota in maternal stress and maternal separation in brain and associated behaviour [14]. Prenatal stress has been shown to change the composition of the microbiome in adult rat [15] neonatal mice [16] and infant humans [17]. Changes in the gut microbiota composition were reported in monkeys subjected to maternal separation between six and nine months of age with shedding of lactobacilli three days following separation, followed by the return of normal lactobacilli levels seven days later. Moreover, we have previously shown, albeit using somewhat crude Denaturing Gradient Gel Electrophoresis- based analysis, that adult rats that underwent maternal separation showed altered faecal microbial composition compared with normally reared control animals [4].

It is well recognized that eating habits are of relevance to (mental) health [18]. Moreover, there is a growing appreciation for the impact of dietary fatty acids on the intestinal microbiota composition of the host [19–21]. Being critical in the development and function of the CNS, n-3 polyunsaturated fatty acids (n-3 PUFAs) have been under the spotlight for decades [22]. The possible underlying mechanisms by which n-3 PUFAs exert their beneficial effects on health are diverse, involving for instance, HPA, neuroendocrine and immune regulations [23, 24].

In light of these observations, we reported recently the beneficial effects of n-3 PUFAs on reduction of anxiety-like, depressive-like behaviours and improved cognition in female rats [25]. Here we hypothesize that these beneficial effects of long-term intake of n-3 PUFAs would have an impact on intestinal microbiota populations, which in turn, would contribute to the reverse of gut-brain axis dysfunction associated with maternal separation. To the best of our knowledge, this is the first study to describe the impact of n-3 PUFAs on the gut-microbiota of female rats exposed to early-life stress.

## Materials and Methods

### Maternal separation

Animals were provided by Biological Services Unit (BSU), UCC, Cork, Ireland. All scientific procedures were carried out in line with Directive 2010/63/EU and were approved by the Animal Experimentation Ethics Committee of University College Cork #2012/036. Maternal separation was performed as previously described by our group [25, 26]. Briefly, male and female rats were obtained from Harlan Laboratories UK (250–300 g) and mated in the local animal unit. Food and water was available *ad libitum* and animals were maintained on a 12:12-h dark–light cycle with temperature at 20 ± 1°C. MS animals were separated from their mothers from postnatal day (PND) 2 to 12, for three hours a day. Separations were conducted between 0900h and 1200h a.m. in plastic cages placed on top of heater pads (30–33°C) in a separate room to the main holding room. Non-separated (NS) animals were left undisturbed in their home cages with their respective dams and were returned to the holding room. After postnatal day 12, rats were left undisturbed except for routine cage cleaning every two days and a weekly body weight measurement until they were 5 weeks old. Animals were group-housed 5 per cage in plastic cages with sawdust bedding in an enriched environment with shredded paper and a cardboard roll.

### Treatments

**Fig 1:**
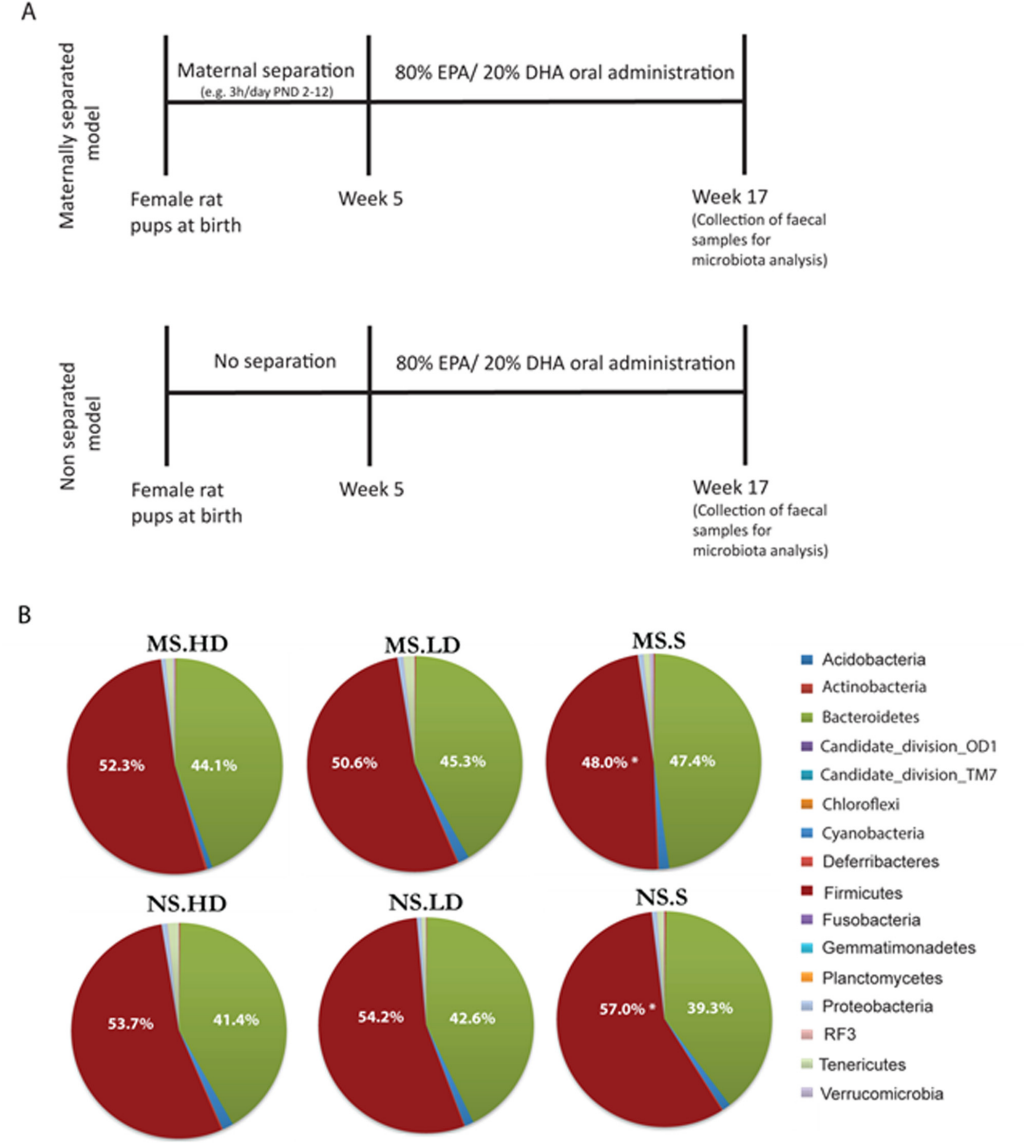
Schematic representation of the time course of the maternal separation procedure and EPA/DHA treatment. (B) Global average microbial composition of faecal 17 weeks old rats samples (n = 10 per group) at phylum-level. * Indicate bacterial group significantly different between MS and NS groups.

Oral administration of an eicosapentaenoic acid (EPA)/docosaexaenoic acid (DHA) (80% EPA, 20% DHA) n-3 PUFAs mixture was administered by gavage when animals reached 5 weeks of age. In order to avoid any confounding litter effects, individual groups consisted of rats from multiple litters. Treatments consisted of 1) saline water; 2) EPA/DHA 0.4 g/kg/day or Low Dose (LD); 3) EPA/DHA 1 g/kg/day or High Dose (HD). The chosen EPA/DHA concentrations were based on the Food and Agriculture Organization of the United Nations (FAO) recommendations. FAO recommends a minimum DHA intake of 10–12 mg/kg per body weight for children 6–24 months old and EPA/DHA 100–250 mg/day for children aged 2–10 years. In our study, the maximum EPA/DHA intake was 100 and 250 mg for the low dose and the high dose per body rat, respectively. Moreover, previous studies have used the same concentrations proposed in this study [27, 28]. Treatments were prepared freshly every day and administered between 0900h and 1100h a.m. The experimental time line is shown in Fig 1A.

### Sample collection

Faecal pellets were collected from 17 weeks old female rats. All samples were, then, frozen at −80°C for microbiota analysis.

### Microbiota analysis

DNA was extracted using the DNA Fast Stool DNA extraction kit (Qiagen) using the protocol for Gram positive bacteria and including an additional bead beating step at the beginning of the procedure. DNA was quantified using the Qubit High Sensitivity Kit (Life Technologies), standardised and then used as a template for PCR. PCR primers and conditions are essentially as outlined in the Illumina 16S Metagenomic Sequencing Library preparation guide (Illumina) with the following exceptions: For the initial 16S PCR, the PCR was performed in duplicate 50 ul reaction volumes, and 40 cycles were used in the PCR. Products were then pooled, cleaned with an appropriate volume of Ampure beads and eluted in 30ul/sample. This was then used as the template for the index PCRs as outlined in the protocol (Illumina). Library quantification, normalisation, and pooling were as outlined in the protocol. After pooling, the sample was requantified using the Qubit High Sensitivity Kit (Life Technologies) and run on an agilent high sensitivity chip (Agilent). Library denaturation and MiSeq sample loading were then performed as described in the protocol. The final concentration of the library was 4pM and PhiX was spiked in as a control at 5% v/v. A 2 x 300bp MiSeq reagent was used for sequencing. Diversity analyses were performed in QIIME and correlations used the websites Calypso at http://bioinfo.qimr.edu.au/calypso.

## Results and Discussion

### Long-term supplementation of EPA/DHA restores the microbiota composition in the MS rats

In this study, the microbial composition of the faecal samples collected from MS and NS EPA/DHA treated and saline rats revealed significant changes in the relative abundance of the main dominant phyla; Bacteroidetes and Firmicutes between the MS-saline and NS-saline (Fig 1B). These data suggest a state of microbial dysbiosis in MS-saline group. The results coincide with our recent findings that early life stress was associated with induced inflammatory cytokines in plasma [25]. Indeed, a recent study using bacterial tag encoded FLX amplicon pyrosequencing demonstrated that repeated social stress, associated with elevated levels of inflammatory cytokines decreased the relative abundance in cecal bacteria of the genus Bacteroidetes, while increasing the relative abundance of bacteria in the genus Firmicutes [29]. Moreover, reduced Bacteroidetes: Firmicutes ratio in human stool specimen has also been shown in depressed individuals as well as in irritable bowel syndrome (IBS) patients, which is often accompanied by depressive symptoms [30, 31]. Interestingly, long-term administration of EPA/DHA reversed the early-life stress-induced Bacteroidetes: Firmicutes shift in MS adult rats (Fig 1B). Presumably, this shift suggests an anti-inflammatory effect [32] of EPA/DHA supplementation, as we previously reported [25].

### Long-term EPA/DHA supplementation shifts the microbiota composition in MS rats towards a profile similar to that in NS rats regardless of the dose

**Fig 2:**
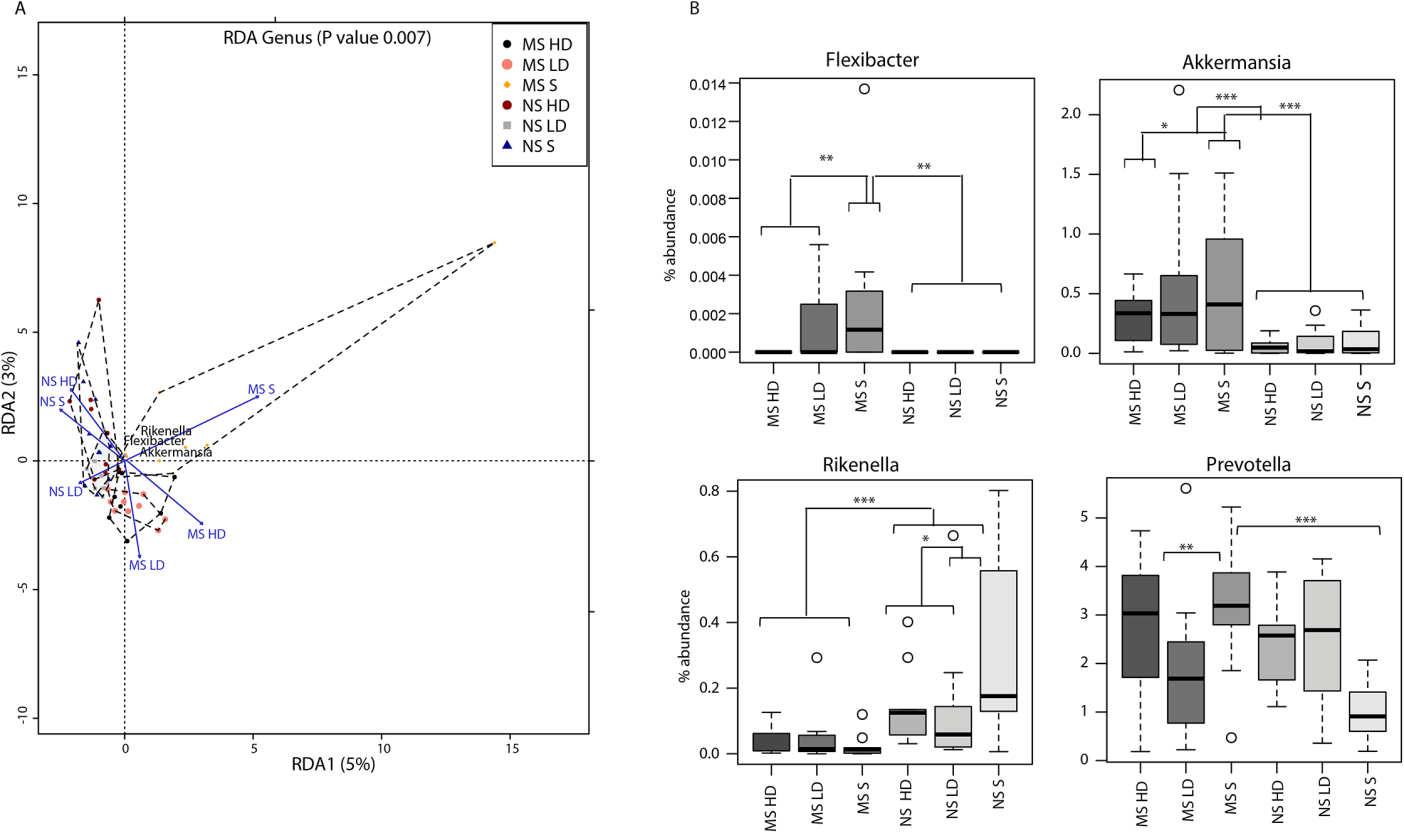
(A) Redundancy analysis (RDA) based on the genus level showing a significant separation between the EPA/DHA treated and saline MS groups (P = 0.007). The hulls identify the centroids of each dataset. (B) Relative abundance of selected genera in the MS and NS saline and EPA/DHA treated groups.

The difference of the global microbiota composition from the 16S rDNA data of the six groups was assessed by ordination (Fig 2A). Statistics based on random permutations of the redundancy analysis (RDA) showed that the MS-saline group can significantly be separated at genus level (p<0.001) from the MS EPA/DHA treated groups and the NS-saline and EPA/DHA treated groups. The centroids of the MS-saline and NS groups were clearly separated; whereas the MS EPA/DHA treated groups were in an intermediate position between the MS-saline and NS groups. Long-term supplementation of low dose of EPA/DHA in NS rats appears to have a different impact on the microbiota composition when compared to the NS-saline and NS-HD groups. Together, the results point to possible interactions between EPA/DHA and members of the gut microbiota, which may eventually influence their biological roles. In fact, *in vitro* interactions of PUFAs with some probiotics have been shown to affect the growth and adhesion of different Lactobacillus strains [33].

Statistical analyses of the differences of microbiota composition among the six groups at genus level identified several taxa differentially present between the MS, NS EPA/DHA treated and saline rats (Table 1). The significance of the taxa *Akkermansia*, *Rikenella*, *Prevotella*, and *Flexibacter* are among the main discriminants between MS-saline, MS EPA/DHA treated and NS groups (Fig 2B). The relative abundance of *Akkermansia* was induced by maternal separation suggesting elevated inflammatory response, as we recently reported [25]. In an inflammatory milieu, *Akkermansia*, the mucus degrader, facilitates the microbial translocation to come in direct contact with the intestinal epithelium, therefore exacerbating gut inflammation [34, 35]. The reduced abundance of Akkermansia in EPA/DHA treated groups coincide with the findings reporting that high concentrations of PUFAs inhibited growth and adhesion to mucus of several bacterial strains [33]. Similarly, *Flexibacter*, a member of Bacteroidetes, was elevated in the microbiota isolated from tissue specimens of a subset of patient with Crohn’s disease and ulcerative colitis [36]. *Prevotella* was shown to increase the sensitivity to chemically induced colitis in experimental mice [37]. Collectively, the data reveals that *taxa*, previously reported to be associated with a state of inflammation, are significantly more abundant in the gut of MS rats. Long-term n-3 PUFAs supplementation appears to restore the microbial balance to a state similar to that in NS rats. Therefore, it is tempting to speculate a possible EPA/DHA anti-inflammatory effect through the regulation of the gut microbiota composition.

**Table 1:** Significant differentially abundant taxa between MS and NS EPA/DHA treated and saline groups as calculated by Wilcoxon rank test at genus level, indicated by the p-value.

Taxa	P	FDR qValue	Median NS S	Median NS LD	Median NS HD	Median MS S	Median MS LD	Median MS HD
Planctomycetes-Phycisphaerae- WD2101	0.00045	0.0319	0	0	0	0.00484	0.00325	0.011374
Rikenellaceae-Rikenella	0.00052	0.0319	0.175714	0.058656	0.124997	0.012741	0.014224	0.032709
Acidobacteria- Acidobacteria_bacterium	0.00066	0.0319	0	0	0	0.002806	0.002105	0.001315
Aerococcaceae-Aerococcus	0.0013	0.047125	0	0	0	0	0	0.001569
Cytophagaceae-Flexibacte	0.0018	0.0522	0	0	0	0.001165	0	0
Burkholderiaceae-Cupriavidus	0.0025	0.059813	0	0	0	0.002325	0	0
Oxalobacteraceae-Massilia	0.0029	0.059813	0	0	0	0.000695	0	0
Xanthomonadaceae-Rhodanobacter	0.0033	0.059813	0	0	0	0	0	0.00215
Planctomycetaceae-Planctomyces	0.0053	0.076962	0	0	0	0.001545	0	0.00052
Chitinophagaceae-uncultured	0.0057	0.076962	0	0	0	0	0	0.00131
Acidobacteria-DA023- uncultured_bacterium	0.0059	0.076962	0	0	0	0.001602	0	0.000259
Acidobacteria-DA023- uncultured_bacterium	0.0067	0.076962	0	0	0.000315	0.001149	0	0.00271
Verrucomicrobiaceae-Akkermansia	0.0069	0.076962	0.034586	0.017324	0.048543	0.41013	0.330376	0.336236
Mollicutes-RF9-uncultured	0.0082	0.080233	0.257038	0.334292	0.392858	0.249543	0.21356	0.114447
Acidobacteriaceae-Candidatus	0.0083	0.080233	0	0	0	0.00291	0	0
Prevotellaceae-Prevotella	0.0092	0.083375	0.9102	2.68956	2.576339	3.190481	1.688466	3.033729
Spartobacteria-Chthoniobacterales	0.025	0.1885	0	0	0	0.00914	0.006015	0.00289
Caldicoprobacteraceae- Caldicoprobacter	0.026	0.1885	0.019758	0.010302	0.012136	0.00734	0.00214	0.002545
Rikenellaceae-Alistipes	0.037	0.2262	0	0	0	0	0	0.000525
Clostridiaceae-Clostridium	0.039	0.2262	0.167448	0.154021	0.194113	0.13282	0.086339	0.103405
Ruminococcaceae- uncultured_bacterium	0.039	0.2262	0.001072	0.00103	0.000944	0	0	0
Bacillaceae-Bacillus	0.043	0.239808	0	0	0	0.000396	0	0

Values for the six groups are medians of the relative abundance of the indicated genus. The FDR q-values are adjusted p-values that correct for multiple testing at a defined false discovery rate (Benjamini et al., 1995).

### Low corticosterone levels in MS rats is correlated with Akkermansia and Rikenella

**Fig 3:**
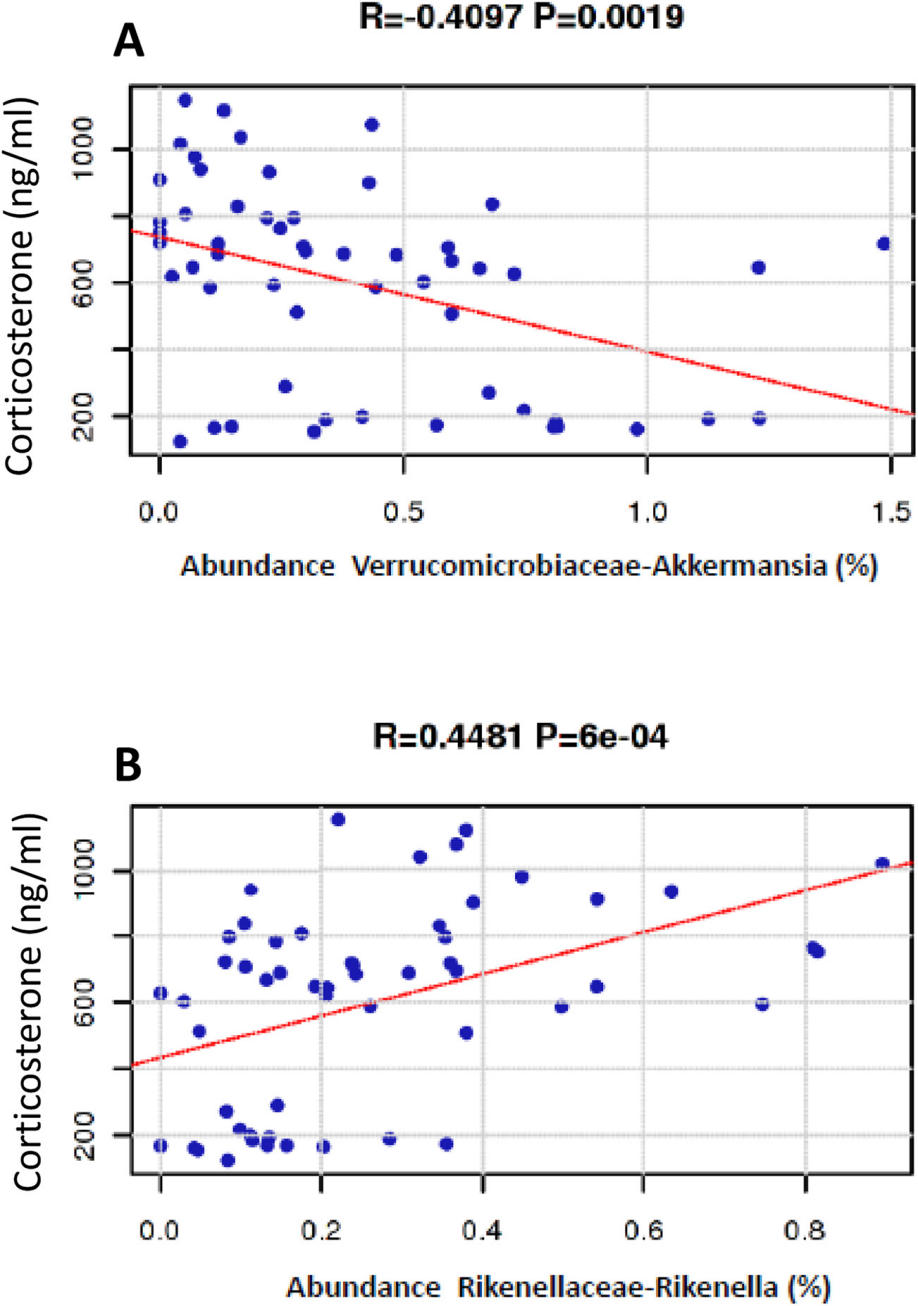
Correlation between percentage abundance of *Akkermansia* and *Rikenella* and corticosterone (CORT) plasma levels. Regression analysis revealed (A) negative correlation between low CORT levels in MS-saline group and low abundance of *Akkermansia* and (B) positive correlation between low CORT levels in MS-saline group and low abundance of *Rikenella*.

Recently, we showed that stress-induced corticosterone levels were reduced in MS group compared to the respective controls when exposed to acute stress [25]. In order to investigate a possible correlation between the altered microbiota in the gut of MS-saline group and the HPA, regression analysis was performed between corticosterone levels and the gut microbiota of MS and NS groups. Interestingly, a negative correlation with *Akkermansia* (R = 0.4097, P = 0.0019) and positive correlation with *Rikenella* (R = 0.4481, P = 6e-04) was observed (Fig 3). While *Rikenella* was reported to be associated with reduced risk of colitis [38](Couturier-Maillard, J Clin Invest. 2013 Feb 1; 123(2): 700–711), *Akkermansia* has been previously shown to exacerbate gut inflammation in mice [34, 35]. Thus, we consider that the observed high *Akkermansia* abundance in MS rats may contribute to elevated levels of inflammation, which in turn may activate the HPA as recently shown [25]. In view of that, persistent activation of HPA may eventually lead to end organ burnout and consequent lower CORT release. Accordingly, HPA hypoactivity has been previously reported in other animal models of stress [39–41].

### Effects of long-term EPA/DHA supplementation on the composition of the gut microbiota is more pronounced in MS rats

**Fig 4:**
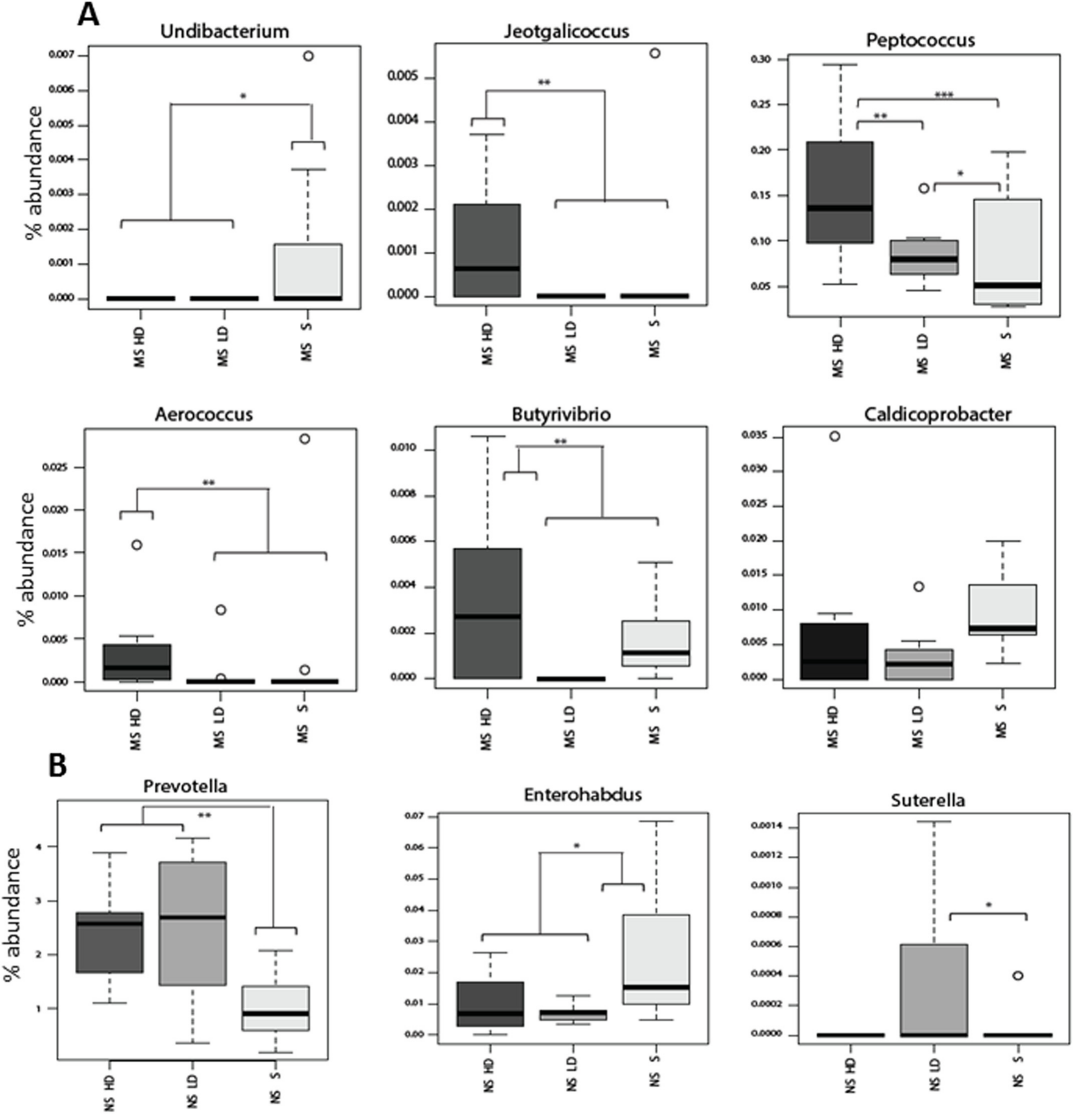
Effects of maternal separation (MS) and EPA/DHA administration on rat gut microbiota. Relative abundance of significantly altered microbial genera in the MS-saline and EPA/DHA treated groups (A) and NS-saline and EPA/DHA treated groups (B). Median with interquartile ranges is depicted. Significant difference indicated by *, p<0.05; **, p<0.01; ***, p<0.001.

Long-term EPA/DHA supplementation has a strong impact on the composition of the gut microbiota in MS rats (Fig 4A). In particular, high dose administration of EPA/DHA was associated with higher levels of the butyrate producing bacteria; Butyrivibrio. Moreover, high dose supplementation of EPA/DHA elevated the levels of several members Actinobacteria (such as *Aerococcus*), with a concomitant reduction of the abundance of members of Proteobacteria (such as *Undibacterium*). The results are in agreement with a recent study on maternal prenatal stress, which reported lower quantities of lactic acid bacteria such as Lactobacillus, Lactococcus, Aerococcus and Bifidobacteria and significantly higher relative abundance of Proteobacterial members [17]. Altogether, this pattern of altered gut microbiota in MS rats support the existence of a potentially increased level of inflammation, which could be reversed by long-term supplementation of high dose of n-3 PUFAs. In fact, diets rich in PUFAs have been shown to positively influence immune function [42]. The possible underlying mechanisms by which PUFAs exert their beneficial effects on health were shown to involve the inhibition of pro-inflammatory cytokine synthesis (tumor necrosis factor alpha and interleukin-1), modulation of the hypothalamic-pituitary-adrenal anti-inflammatory responses, and induction of the release of acetylcholine [43].

Long-term EPA/DHA supplementation in NS rats had less significant impact on their gut microbiota in comparison to their effect observed in MS rats (Fig 4B). In NS rats, long-term administration of EPA/DHA was associated with changes in the abundance of three taxa; *Enterorhabdus*, *Sutterella*, and *Prevotella* (Fig 3B). *Sutterella* was shown to be associated with some gastrointestinal infections in humans [44]. Moreover, *Sutterella* was shown to be of significantly higher prevalence in biopsies taken from the gut of autistic children with gastrointestinal disturbance compared to controls with GI disturbance [45]. Together, our data supports the expected beneficial effects of n-3 PUFAs particularly in a state of microbial dysbiosis, which is associated with inflammation.

## Conclusion

We demonstrate what is to our knowledge the first time that EPA/DHA treatment normalized early-life stress-induced disruption of female rat gut microbes. Analysis of the gut microbiota of MS rats showed altered microbial composition with abundance of members previously shown to be associated with inflammation. These results indicate that transient stress-induced alteration during a crucial developmental time-window for neonatal rats has long-lasting effects on the gut microbiota composition in adulthood. Supplementation with EPA/DHA restored the composition of the gut microbiota in MS rats. Presumably, the EPA/DHA effect on the gut microbiota is related to PUFAs anti-inflammatory activity. Recently, it has been shown that omega-3 and omega-6 dramatically reduce the endotoximic and inflammatory status in metabolic endotoxemia (Kaliannan, 2015). Intriguingly, the observed effects involved changes in the gut microbiota, through the impact of omega-6, -3 on intestinal production and secretion of intestinal alikaline phosphatase. The induced alterations in gut microbiota composition resulted in reduction in levels of lipopolysachharides and gut permeability, which in turn reduces the onset of inflammation.

Overall, the healthy benefits at CNS level have ascertained the contribution made by n-3 PUFAs to stress-related disorders and put them under the spotlight for decades [46–48]. The current study offers insight into a potential role of n-3 PUFAs through the modification of the gut microbiota in an animal model of stress. Our data supports the previous reports showing that the absence as well as the exacerbation of certain bacterial taxa in the gut of early-life stressed rats may represent risk factors for the development of anxiety, depression and inflammatory diseases such as IBS. We postulate that EPA/DHA administration is beneficial for restoring members of the microbiota with immunoregulatory functions in order to prevent an overly robust stress-induced inflammatory response which may contribute to the onset of mental illnesses.

## Supporting Information

S1 File Microbiota Data Set. NS.S, NS.LD, NS.HD stand for non-separated Saline, non-separated Low Dose, non-separated High Dose, respectively. MS.S, MS.LD, MS.HD stand for maternally separated Saline, maternally separated Low Dose, maternally separated High Dose, respectively.
(ZIP)
