# Correction: N-3 Polyunsaturated Fatty Acids (PUFAs) Reverse the Impact of Early-Life Stress on the Gut Microbiota

**DOI:** 10.1371/journal.pone.0142228

**Published:** 2015-10-30

**Authors:** Matteo M. Pusceddu, Sahar El Aidy, Fiona Crispie, Orla O’Sullivan, Paul Cotter, Catherine Stanton, Philip Kelly, John F. Cryan, Timothy G. Dinan

The affiliation for the second author is missing. Along with #2, Sahar El Aidy is also affiliated with: Microbial Physiology, Groningen Biomolecular Sciences and Biotechnology Institute, University of Groningen, Groningen, The Netherlands
