# Supplementary material for: N-3 Polyunsaturated Fatty Acids (PUFAs) Reverse the Impact of Early-Life Stress on the Gut Microbiota
Source: PLoS One. 2015 Oct 1;10(10):e0139721. doi: 10.1371/journal.pone.0139721 (PMC4591340; doi:10.1371/journal.pone.0139721)
Supplement: S1 File — NS.S, NS.LD, NS.HD stand for non-separated Saline, non-separated Low Dose, non-separated High Dose, respectively. MS.S, MS.LD, MS.HD stand for maternally separated Saline, maternally separated Low Dose, maternally separated High Dose, respectively. (ZIP) [file pone.0139721.s001.zip › Matteo/unweighted_unifrac_emperor/index.html]

Emperor


|  |
| --- |
|  |


# WebGL is not enabled!

Emperor's visualization framework is WebGL based, it seems that your system doesn't have this resource available. Here is what you can do:

**Chrome:** Type "chrome://flags/" into the address bar, then search for "Disable WebGL". Disable this option if you haven't already. *Note:* If you follow these steps and still don't see an image, go to "chrome://flags/" and then search for "Override software rendering list" and enable this option.

**Safari:** Open Safari's menu and select Preferences. Click on the advanced tab, and then check "Show Developer" menu. Then open the "Developer" menu and select "Enable WebGL".

**Firefox:** Go to Options through Firefox > Options or Tools > Options. Go to Advanced, then General. Check "Use hardware acceleration when available" and restart Firefox.

**Other browsers:** The only browsers that support WebGL are Chrome, Safari, and Firefox. Please switch to these browsers when using Emperor.

*Note:* Once you went through these changes, reload the page and it should work!

Sources: Instructions for Chrome and Safari, and Firefox

PCoA
Parallel

- Key
- Colors
- Visibility
- Scaling
- Labels
- Axes
- Options

Filter

Use gradient colors

|  |
| --- |
|  |
|  |
| Global Sphere Opacity |

|  |
| --- |
|  |
|  |
| Global Sphere Scale |

Samples Label Visibility
  
Label Opacity


|  |  |
| --- | --- |
|  | Master Label Color |

|  |  |
| --- | --- |
|  | Axes Labels Color |
|  | Axes Color |
|  | Background Color |

Scale coords by percent explained

  
  
  


---

  
Filename (only letters, numbers, ., - and \_):
  
  
 Create legend
  
  
For a PNG, simply press 'ctrl+p'.
