# Supplementary figures and images for: N-3 Polyunsaturated Fatty Acids (PUFAs) Reverse the Impact of Early-Life Stress on the Gut Microbiota
Source: PLoS One. 2015 Oct 1;10(10):e0139721. doi: 10.1371/journal.pone.0139721 (PMC4591340; doi:10.1371/journal.pone.0139721)

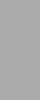

Supplement: S1 File — NS.S, NS.LD, NS.HD stand for non-separated Saline, non-separated Low Dose, non-separated High Dose, respectively. MS.S, MS.LD, MS.HD stand for maternally separated Saline, maternally separated Low Dose, maternally separated High Dose, respectively. (ZIP) [file pone.0139721.s001.zip › Matteo/unweighted_unifrac_emperor/emperor_required_resources/css/images/ui-bg_flat_0_aaaaaa_40x100.png]

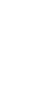

Supplement: S1 File — NS.S, NS.LD, NS.HD stand for non-separated Saline, non-separated Low Dose, non-separated High Dose, respectively. MS.S, MS.LD, MS.HD stand for maternally separated Saline, maternally separated Low Dose, maternally separated High Dose, respectively. (ZIP) [file pone.0139721.s001.zip › Matteo/unweighted_unifrac_emperor/emperor_required_resources/css/images/ui-bg_flat_75_ffffff_40x100.png]

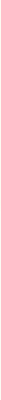

Supplement: S1 File — NS.S, NS.LD, NS.HD stand for non-separated Saline, non-separated Low Dose, non-separated High Dose, respectively. MS.S, MS.LD, MS.HD stand for maternally separated Saline, maternally separated Low Dose, maternally separated High Dose, respectively. (ZIP) [file pone.0139721.s001.zip › Matteo/unweighted_unifrac_emperor/emperor_required_resources/css/images/ui-bg_glass_55_fbf9ee_1x400.png]

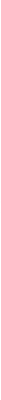

Supplement: S1 File — NS.S, NS.LD, NS.HD stand for non-separated Saline, non-separated Low Dose, non-separated High Dose, respectively. MS.S, MS.LD, MS.HD stand for maternally separated Saline, maternally separated Low Dose, maternally separated High Dose, respectively. (ZIP) [file pone.0139721.s001.zip › Matteo/unweighted_unifrac_emperor/emperor_required_resources/css/images/ui-bg_glass_65_ffffff_1x400.png]

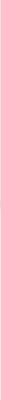

Supplement: S1 File — NS.S, NS.LD, NS.HD stand for non-separated Saline, non-separated Low Dose, non-separated High Dose, respectively. MS.S, MS.LD, MS.HD stand for maternally separated Saline, maternally separated Low Dose, maternally separated High Dose, respectively. (ZIP) [file pone.0139721.s001.zip › Matteo/unweighted_unifrac_emperor/emperor_required_resources/css/images/ui-bg_glass_75_dadada_1x400.png]

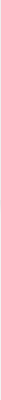

Supplement: S1 File — NS.S, NS.LD, NS.HD stand for non-separated Saline, non-separated Low Dose, non-separated High Dose, respectively. MS.S, MS.LD, MS.HD stand for maternally separated Saline, maternally separated Low Dose, maternally separated High Dose, respectively. (ZIP) [file pone.0139721.s001.zip › Matteo/unweighted_unifrac_emperor/emperor_required_resources/css/images/ui-bg_glass_75_e6e6e6_1x400.png]

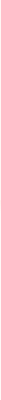

Supplement: S1 File — NS.S, NS.LD, NS.HD stand for non-separated Saline, non-separated Low Dose, non-separated High Dose, respectively. MS.S, MS.LD, MS.HD stand for maternally separated Saline, maternally separated Low Dose, maternally separated High Dose, respectively. (ZIP) [file pone.0139721.s001.zip › Matteo/unweighted_unifrac_emperor/emperor_required_resources/css/images/ui-bg_glass_95_fef1ec_1x400.png]

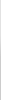

Supplement: S1 File — NS.S, NS.LD, NS.HD stand for non-separated Saline, non-separated Low Dose, non-separated High Dose, respectively. MS.S, MS.LD, MS.HD stand for maternally separated Saline, maternally separated Low Dose, maternally separated High Dose, respectively. (ZIP) [file pone.0139721.s001.zip › Matteo/unweighted_unifrac_emperor/emperor_required_resources/css/images/ui-bg_highlight-soft_75_cccccc_1x100.png]

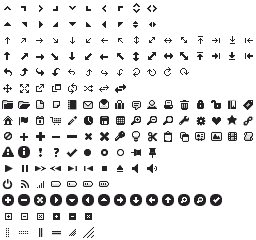

Supplement: S1 File — NS.S, NS.LD, NS.HD stand for non-separated Saline, non-separated Low Dose, non-separated High Dose, respectively. MS.S, MS.LD, MS.HD stand for maternally separated Saline, maternally separated Low Dose, maternally separated High Dose, respectively. (ZIP) [file pone.0139721.s001.zip › Matteo/unweighted_unifrac_emperor/emperor_required_resources/css/images/ui-icons_222222_256x240.png]

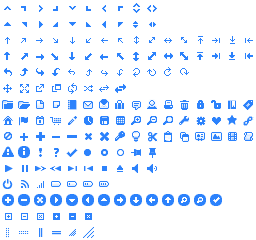

Supplement: S1 File — NS.S, NS.LD, NS.HD stand for non-separated Saline, non-separated Low Dose, non-separated High Dose, respectively. MS.S, MS.LD, MS.HD stand for maternally separated Saline, maternally separated Low Dose, maternally separated High Dose, respectively. (ZIP) [file pone.0139721.s001.zip › Matteo/unweighted_unifrac_emperor/emperor_required_resources/css/images/ui-icons_2e83ff_256x240.png]

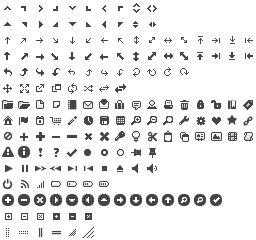

Supplement: S1 File — NS.S, NS.LD, NS.HD stand for non-separated Saline, non-separated Low Dose, non-separated High Dose, respectively. MS.S, MS.LD, MS.HD stand for maternally separated Saline, maternally separated Low Dose, maternally separated High Dose, respectively. (ZIP) [file pone.0139721.s001.zip › Matteo/unweighted_unifrac_emperor/emperor_required_resources/css/images/ui-icons_454545_256x240.png]

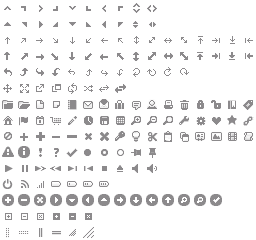

Supplement: S1 File — NS.S, NS.LD, NS.HD stand for non-separated Saline, non-separated Low Dose, non-separated High Dose, respectively. MS.S, MS.LD, MS.HD stand for maternally separated Saline, maternally separated Low Dose, maternally separated High Dose, respectively. (ZIP) [file pone.0139721.s001.zip › Matteo/unweighted_unifrac_emperor/emperor_required_resources/css/images/ui-icons_888888_256x240.png]

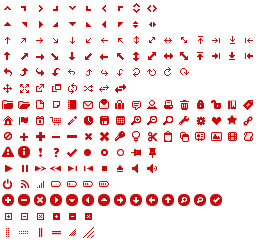

Supplement: S1 File — NS.S, NS.LD, NS.HD stand for non-separated Saline, non-separated Low Dose, non-separated High Dose, respectively. MS.S, MS.LD, MS.HD stand for maternally separated Saline, maternally separated Low Dose, maternally separated High Dose, respectively. (ZIP) [file pone.0139721.s001.zip › Matteo/unweighted_unifrac_emperor/emperor_required_resources/css/images/ui-icons_cd0a0a_256x240.png]

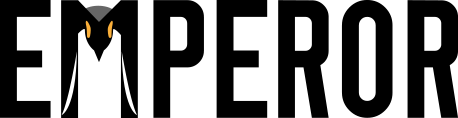

Supplement: S1 File — NS.S, NS.LD, NS.HD stand for non-separated Saline, non-separated Low Dose, non-separated High Dose, respectively. MS.S, MS.LD, MS.HD stand for maternally separated Saline, maternally separated Low Dose, maternally separated High Dose, respectively. (ZIP) [file pone.0139721.s001.zip › Matteo/unweighted_unifrac_emperor/emperor_required_resources/img/emperor.png]

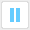

Supplement: S1 File — NS.S, NS.LD, NS.HD stand for non-separated Saline, non-separated Low Dose, non-separated High Dose, respectively. MS.S, MS.LD, MS.HD stand for maternally separated Saline, maternally separated Low Dose, maternally separated High Dose, respectively. (ZIP) [file pone.0139721.s001.zip › Matteo/unweighted_unifrac_emperor/emperor_required_resources/img/pause.png]

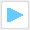

Supplement: S1 File — NS.S, NS.LD, NS.HD stand for non-separated Saline, non-separated Low Dose, non-separated High Dose, respectively. MS.S, MS.LD, MS.HD stand for maternally separated Saline, maternally separated Low Dose, maternally separated High Dose, respectively. (ZIP) [file pone.0139721.s001.zip › Matteo/unweighted_unifrac_emperor/emperor_required_resources/img/play.png]

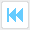

Supplement: S1 File — NS.S, NS.LD, NS.HD stand for non-separated Saline, non-separated Low Dose, non-separated High Dose, respectively. MS.S, MS.LD, MS.HD stand for maternally separated Saline, maternally separated Low Dose, maternally separated High Dose, respectively. (ZIP) [file pone.0139721.s001.zip › Matteo/unweighted_unifrac_emperor/emperor_required_resources/img/reset.png]
